# A safety assessment of *Coriolus versicolor* biomass as a food supplement

**DOI:** 10.3402/fnr.v60.29953

**Published:** 2016-03-10

**Authors:** Ana B. Barros, Jorge Ferrão, Tito Fernandes

**Affiliations:** 1 Global Health and Tropical Medicine, Institute of Hygiene and Tropical Medicine, New University of Lisbon, Lisbon, Portugal; 2 Ministry of Education, Maputo, Mozambique; 3 Faculty of Veterinary Medicine, Lisbon University, Lisbon, Portugal

**Keywords:** Coriolus versicolor, biomass, food supplement, safety assessment, mushrooms

## Abstract

**Background:**

*Coriolus versicolor* (CV) is a common mushroom with antitumor, anti-inflammatory, antioxidant, antiviral, antibacterial, and immunomodulatory properties. The existence of these properties has been extensively proven mainly using CV extract; research on the biomass form is scarce.

**Objective:**

The aim of this study was to investigate the safety of the CV biomass form, as it is commonly used as a food supplement.

**Design:**

CV biomass powder was dissolved in distilled water and administered daily (2.5, 5.0, and 7.5 g/kg live weight) in single doses by gavage to both female and male Charles River albino rats.

**Results:**

No adverse or lethal effects were observed as a consequence of the daily administration of CV biomass. In addition, compared with the control group, no abnormal findings were observed at necropsy and histopathological examination.

**Conclusions:**

A safe profile of CV biomass for human consumption can be inferred from the absence of any remarkable adverse effects in rats.

Mushrooms have an established history of human use for thousands of years in traditional oriental therapies (1). Modern clinical practice in Japan, China, Korea, and other Asian countries continues to depend on mushroom-derived preparations. *Coriolus versicolor* (CV; also known as *Trametes versicolor*) is one of the most common mushrooms; it can be found just about anywhere there are decomposing hardwood logs or tree stumps and is used for its nutrition and health promoting properties (2). Numerous studies have demonstrated the antitumor, anti-inflammatory, antioxidant, antiviral, antibacterial, and immunomodulatory activities of mushrooms (3–7). Mushrooms such as CV increase the activity of lymphocytes – B-cells, T-cells, and especially natural killer (NK) cells. B-cells produce antibodies, whereas T-cells and NK cells destroy virally or bacterially infected cells and cells that have turned cancerous (8). CV can be used in two forms: extract and biomass. CV extract has been extensively studied in several research articles (9–11), and its biological material consists of the concentrated extract of the fruiting bodies. CV biomass contains mycelia and primordia and is more resistant to proteolytic enzymes (i.e. under stimulation of the digestive tract) than the extract form (12). The biomass form also incorporates important immune-enhancing enzyme activity, such as superoxide dismutase, peroxidase, glucoamylase, and protease activities, which are not detected in the extracted forms of mushrooms. In recent years, much research has focused on characterising the physiological effects resulting from human consumption of a wide variety of dietary fibre sources (13). The biomass form of mushrooms provides not only β-glucans (e.g. lentinan, schizophyllan, and grifolan), also supplied in the extract form, but also important immune-enhancing enzyme activity (e.g. cytochrome P-450, cytochrome reductase, peroxidase, glucoamylase, β-glucanase, glucose 2-oxidase, laccase, superoxide dismutase, and protease) and secondary metabolites (e.g. terpenes, steroids, anthraquinones, benzoic acid derivatives, and quinolones) that are not detected in significant quantities in the extracted forms of mushrooms (14). Therefore, the biomass form of mushrooms is considered more beneficial in promoting detoxification and preventing oxidative stress and cellular growth (15). The properties, physiological activity, recovery, and purification of the bioactive polysaccharopeptides (PSP) of CV extract have been extensively reviewed (16–26). Of the mushroom-derived immunonutrients, proteoglycans (polysaccharopeptide Krestin and PSP) obtained from extracted CV are commercially the best established as prebiotics (27). While accessible for the extracted form, to date no study was found available in the literature on the safety profile of CV biomass as a food supplement. Thus, the purpose of this study was to produce solid scientific evidence regarding the safety assessment of CV biomass with regard to extrapolating the data to humans according to international guidelines. In order to achieve this goal, the present study was conducted with laboratory animals Charles River albino rats, strain 273 (IGS Programme), using different and increasing levels of inclusion of CV biomass, doses related to animal weight and calculated from previous knowledge of administration in humans. The study complies with EU guidelines (28–31).

## International regulatory aspects

CV biomass is considered a ‘dietary supplement’, a product that contains nutrients derived from food products that are concentrated in liquid or capsule form. It is not a ‘functional food’, since these are designed to allow consumers to eat enriched foods close to their natural state, rather than by taking dietary supplements. In the United States, the Dietary Supplement Health and Education Act of 1994 provided the following definition: ‘A dietary supplement is a product taken by mouth that contains a “dietary ingredient” intended to supplement the diet. The “dietary ingredients” in these products may include vitamins, minerals, herbs or other botanicals, amino acids, and substances such as enzymes, organ tissues, glandular, and metabolites’. Dietary supplements can also be extracts or concentrates, and may be found in many forms such as tablets, capsules, soft gels, gel caps, liquids, or powders (32). It is widely accepted that the health-promoting properties of foods are not necessarily due to single components, but rather a few or several active ingredients. This understanding creates a significant paradigm shift from the pharmaceutical model, which is based on the efficacy of single agents. In relation to regulatory purposes and the use of dietary supplements such as CV, regulations differ between countries. Japan was the first country to recognise functional foods as a separate category when in 1991 it introduced a system called *Foods for Specific Health Use* to evaluate health claims based on a list of approved foods and ingredients. The Food and Drug Administration in the United States has changed its stand on health claims and now allows ‘qualified health claims’ for which there is hardly any evidence, as long as a disclaimer is included. Canada, Australia, and New Zealand have introduced new systems to regulate health claims, but experience with these is still limited. The lack of proper regulatory oversight has led to some functional foods and nutraceuticals that are no more than quackery; at the same time, other functional foods do promote health and prevent disease. The potential for effective functional foods and dietary supplements is certainly there (33). The European Commission agreed on new regulations that would prohibit vague claims and that would allow hard claims of disease reduction for foods if the evidence is solid (34). The commission even wants to grant companies 7 years of exclusivity for truly novel claims backed up by solid data. The World Health Organization is also working in this area, hoping to develop an international standard for nutrient profiles that could be used by many countries (35). Therefore advances in knowledge of how micronutrients perturb or otherwise modulate biological processes in the development of diseases or other forms of toxicity will provide the basis for further improving the safety assessment process of dietary supplements.

## Materials and methods

### Experimental samples

CV biomass used in the present trial was purchased from a UK supplier that produces in compliance with good manufacturing practice standards. Mycological products from this supplier are in the form of proprietary biomass cultivated on sterile (autoclaved) substrate, while rigidly conforming to conditions that are applied to the manufacture of conventional pharmaceuticals. Cultivated in California, USA, the result fungal CV biomass is derived from strain number CV-OH1 and certified 100% organic in the United States (Arizona) by Quality Assurance International and qualified as food grade by EU standards in the Netherlands. The material is shipped to the Netherlands, where it is manufactured into a finely ground powder or processed into 500-mg tablets with the addition of cellulose, silica, a granulating agent, and a tablet press lubricant. The finished tablet complies with the British Pharmacopoeia requirements for tablets. A 10-g sample is sent from each batch to a third-party laboratory in Hull, United Kingdom, where PCR DNA and TVAC analysis are performed to confirm the mushroom type and assure that the batch is ‘clean’.

### Experimental animals

Male and female Wistar Han (RccHan: WIST) rats (males, 295–367 g; females, 200–239 g) were obtained from a local dealer (Portugal). The animals were acclimatised to laboratory conditions for 7 days prior to the experiment. During acclimatisation, rats were housed in groups by treatment and sex. There were 4 houses per treatment: 2 houses of 5 male rats each and 2 houses of 5 female rats each. The control group had only 2 houses, one for the 5 female rats and other for the 5 male rats. All animals had free access to tap water. The feed for the experimental animals was purchased from an Italian supplier. The rats were maintained at 25±5°C under a light/dark cycle of 12 h and relative humidity 70±10%. All procedures in this study were performed in accordance with Directive 2010/63/UE and the Portuguese National Regulation (Decree Law 113/2013, of 7 August 2013) and with approval (08/2014) from the Animals Ethics Committee of the Faculty of Veterinary Medicine of Lisbon University.

### Safety assessment

The CV biomass powder was dissolved in distilled water and administered daily (2.5 g, 5.0 g, and 7.5 g/kg live weight/day) in single doses by gavage to both female and male rats (*n*=60; 10 males and 10 females per treatment). In contrast, the control group (*n*=10; 5 males and 5 females) received only the same feed with no addition of CV. The dose of exposure was calculated to ensure a safety factor of 100 corresponding to some 2 g/kg for a 60-kg human individual. The rats were fasted overnight prior to dosing. The general behaviour of the rats was continuously monitored during the 90 days of the experiment and their body weight was measured weekly and their intake adjusted.

At the end of the experiment, all animals were anesthetised with a ketamine hydrochloride/medetomidine hydrochloride combination. Blood samples were collected by cardiac puncture. After blood collection, the rats were sacrificed by clavicle dislocation, and selected vital organs were excised, weighed, and macroscopically examined. The study report included measurements (weighing) and regular detailed observations as well as necropsy procedures and histopathology.

### Statistical analysis

The statistical software SPSS^®^ 22.0 for Mac OS was used to run the analysis. Data are presented as the mean±SEM (standard error of the mean); comparisons among groups were performed by one-way analysis of variance. Significant differences between groups were assessed by Fisher's least significant difference test. *p*-Values less than 0.05 (*p <* 0.05) were considered to be significant.

## Results and discussion

### Main findings and discussion

During the 90 days of the study, a single death was recorded in the control group of male rats at the onset of the trial. Therefore, no lethal effects were observed as a consequence of the daily administration of CV biomass (2.5 g/kg, 5.0 g/kg, and 7.5 g/kg live weight). The appearance and behaviour of the animals were similar in all groups during the 90 days. Normal body weight gains were observed in males and females of all groups and there were no significant differences between relative organ weights (Table 1) of the test groups compared to the control. No abnormal findings were observed at the necropsy. Blood analysis was not performed due to malfunction of storage conditions. A number of histological observations in lungs, intestines, and mandibular lymph nodes were noted in this study but were not related to the administered doses (Table 2). Lung injuries occurred in both the control and test groups, but did not have statistical significance. Intestine and mandibular lymph node lesions were statistically significant, but the control groups of both male and female rats showed more lesions than the treatment groups. In the remaining organs no lesions were identified.

**Table 1:** Relative organ weights (% Organ weight/body weight) of rats that were given *Coriolus versicolor* biomass

	Administered dose							
	
			*Coriolus versicolor* biomass					
			
	Control		2.5 g/kg		5.0 g/kg		7.5 g/kg	
				
Organ	Mean	±SEM	Mean	±SEM	Mean	±SEM	Mean	±SEM
Male								
Bladder	0.066	0.0144	0.046	0.003	0.054	0.004	0.050	0.006
Heart	0.257	0.0053	0.265	0.011	0.265	0.010	0.272	0.009
Kidneys	0.533	0.0300	0.531	0.009	0.539	0.015	0.553	0.012
Liver	2.481	0.0232	2.751	0.081	2.488	0.134	2.805	0.093
Lungs	0.314	0.0102	0.350	0.016	0.429	0.071	0.386	0.035
Spleen	0.163	0.0122	0.160	0.007	0.184	0.011	0.170	0.006
Stomach	0.423	0.0119	0.467	0.014	0.437	0.010	0.431	0.014
Female								
Bladder	0.069	0.003	0.068	0.003	0.071	0.006	0.081	0.006
Heart	0.357	0.013	0.368	0.015	0.362	0.010	0.367	0.007
Kidneys	0.671	0.019	0.680	0.013	0.658	0.016	0.667	0.017
Liver	2.990	0.043	2.965	0.088	3.111	0.079	3.025	0.078
Lungs	0.559	0.048	0.499	0.012	0.505	0.027	0.485	0.015
Spleen	0.283	0.012	0.275	0.010	0.257	0.011	0.251	0.009
Stomach	0.666	0.029	0.666	0.026	0.637	0.017	0.673	0.027

**Table 2:** Proportion of injured organs

	Administered dose				
		
		*Coriolus versicolor* biomass			
			
Organs	Control	2.5 g/kg	5.0 g/kg	7.5 g/kg	*p*
Male					
Lung	0.75	0.6	0.7	0.7	ns
Intestine	1*	0.2	0.5	0.2	<0.05
Mandibular lymph node	0.25*	0	0	0	<0.05
Female					
Lung	0.6	0.8	0.7	0.9	ns
Intestine	0.8**	0.5	0.3	0.1	<0.05

* Male group: The difference in the intestine is significant when compared to the 2.5-g/kg and 7.5-g/kg groups; the difference in the mandibular lymph node is significant when compared to all other groups.

** Female group: The difference is significant when compared to the 7.5-g/kg group. Statistically significant differences were found by one-way analysis of variance followed by least significant difference test; ns = not significant.

Although CV is one of the commonly used medicinal products in Japan and China (36), little toxicological information is available regarding its safety, either for the extract or the biomass forms. A Malaysian study evaluated the potential toxicity of CV utilising a standardised water extract after acute and sub-chronic 28-day administration in rats (10). The authors also did not detect any signs of toxicity in their study. Accumulating evidence suggests that PSP in extract form is nontoxic even when administered at several times the therapeutically effective dosage and over extended periods. Extended use of PSP at 100-fold the normal clinical dose has not induced acute and chronic toxicity in animals and is not teratogenic (17). PSP appears to be safe during pregnancy and no adverse effects of PSP have been observed in female reproductive and embryonic development in mice (37). Chu et al. (16), using CV aqueous extracts, much more concentrated than CV biomass, calculated LD_50_ as 18 g/kg after 90-day administration. According to the classification of Loomis and Hayes (38), substances with LD_50_ between 5 and 15 g/kg are regarded as being virtually non-toxic. As such, in the present study with rats, the no observed adverse effect level (NOAEL) of CV-OH1 biomass was 7.5 g/kg live weight. Therefore the acceptable daily intake of CV-OH1 biomass, using a safety factor of 100, can be established at 4.5 g for a human being of an average weight of 60 kg.

After some exposure to potentially toxic substances, normally there will be a slight reduction in body weight gain and internal organ weights. However, in the repeated dose 90-day oral study, no deaths and no treatment-related signs were observed in animals of any of the groups. All rats at each dosage group continued to gain weight throughout the 90-day study (data not shown), from the juvenile to adult stages (39). Indeed, in the present study, the absolute organ weights in all treated groups of both sexes were not significantly different from those of the control groups. This finding suggests no grossly toxic effect from CV-OH1 biomass.

Although the present trial did not aim at carcinogenicity studies, as described by OECD TG 451 (31), the data obtained here showed no development of neoplastic lesions, therefore indicating a non-potential carcinogenicity effect. Several other experiments involving CV extract showed that the antitumor action is due to the enhancement and potentiation of the cell-mediated immune system through the regulation of immunomodulatory cytokines and activation of the complement system and NK cells (24, 40–42).

### Detailed organ pathology

The pathology laboratory received the formalin-fixed organs of every rat. A reference analysis number was given to each group of animals.

*Heart:* The lesions mentioned are of minor relevance, with no functional interference, and considered not associated with the administration of CV-OH1.

*Lungs:* The high percentage of rats affected by interstitial pneumonia (between 50 and 90% in the various groups), which was particularly severe in the control animals, indicates that this pathology was associated with the conditions of the animal house. It was considered that whenever lungs were affected by interstitial pneumonia up to 20% of the analysed area, there was no interference with the respiratory function. Therefore they were classified as having ‘no relevant changes’ if no other important lesions were detected that could involve loss of respiratory capability, such as granulomas. Granulomas were only identified in the experimental rats, although the percentage of affected lungs was higher in the 5-g/kg group (50% in males and 20% in females). In the animals administered 7.5 g/kg, only 20% of the males were affected. In this same group no females were affected. After evaluating the conditions under which the diet with CV was being administered, it was concluded that these granulomas could result from aspiration of the food with no association with the administration of CV. PAS and Ziehl staining were performed in several lung sections with granulomas to identify possible fungal elements or alcohol-acid-resistant mycobacteria, and both were negative. Marginal emphysema and focal haemorrhages were considered as consequence of the euthanasia.

*Salivary glands:* No significant changes were noted in any group.

*Stomach:* No significant changes were noted in any group.

*Intestine:* These were considered with no significant changes whenever the infiltration of the lamina propria of the mucosa by mononucleated cells was mild, not deforming the villous profile. Whenever only one section of the intestine was moderately affected by infiltration of the lamina propria by mononucleated cells, these were also considered with no significant changes. Desquamation of the surface epithelium, present in both experimental rats and controls, was regarded as a consequence of the delay in fixation of faecal-containing organs in the course of the necropsy procedures. The moderate-to-intense infiltration of the lamina propria of the mucosa by mononucleated cells was considered a relevant change, although it was present in all groups, including controls. The presence of gut-associated lymphoid tissue (GALT) was always considered normal. No cases of GALT hyperplasia were identified. Control rats were generally affected by intestinal inflammation.

*Liver:* Vacuolisation of the hepatocytes was always mild and, although generalized, could be associated with liver glycogen made available when the animals were forced to fast several hours before euthanasia. It has no functional relevance and it is not associated with the administration of CV.

*Pancreas:* No significant changes were noted in any group.

*Spleen:* Hemosiderosis is a common finding in the spleen of adult rats with an increase of pigment within the organ with ageing. The marked congestion mentioned could be a consequence of euthanasia. No variation was found between experimental and controls.

*Mandibular lymph nodes:* No significant changes were noted in any group. The most severe lesion (neutrophilic lymphadenitis) was identified in a control rat. The lymphoid hyperplasia represents the immune system response to antigens and is not considered pathologic, except when leading to a very marked increase in the organ volume and consequently in the number of active lymphoid cells.

*Kidneys:* No significant changes were noted in any group.

The results showed no mortality or signs of toxicity during the chronic toxicity evaluation. In comparison with the controls there were no significant differences in body weight, relative organ weight, gross pathology, or histopathology between the treatment and control groups.

Histopathological investigation of the organs revealed no treatment-related microscopic changes. The histopathological changes recorded were regarded as toxicologically irrelevant because the adverse results did not appear in both sexes, were not treatment- or dose-related, and were not reflected by changes in other related parameters. Additionally, no concurrent changes in the histopathology of the liver or other organs were noted. Moreover, these changes were within normal laboratory range (43) and were considered as incidental, related to the animal laboratory environment when changes were recorded in the lungs or due to the necropsy procedure when inflammation of intestines was recorded. These occurred even more in the control group among both sexes.

## Conclusions

A safety assessment of the test substance as a food supplement was conducted in male and female rats at three different levels of oral inclusion of CV-OH1 biomass. The NOAEL of the biomass for both male and female rats was 7.5 g/kg live weight daily for 90 days. Results demonstrated the safe profile of CV biomass for human consumption, inferred from the absence of any remarkable adverse effect in rats.

Correcting for body surface area increases safety by resulting in a more conservative starting dose estimate. However, the use of a different dose normalisation approach, such as directly equating the human dose to the NOAEL in g/kg, may be appropriate in some circumstances.

Future studies into the mechanisms of action of mushroom biomass and its impact in immunological parameters will be useful to further delineate the interesting roles and properties of various mushrooms in the prevention and treatment of several diseases.
